# Emergent bacterial community properties induce enhanced drought tolerance in *Arabidopsis*

**DOI:** 10.1038/s41522-021-00253-0

**Published:** 2021-11-18

**Authors:** Nan Yang, Joseph Nesme, Henriette Lyng Røder, Xuanji Li, Zhangli Zuo, Morten Petersen, Mette Burmølle, Søren Johannes Sørensen

**Affiliations:** 1 Section of Microbiology, Department of Biology, University of Copenhagen, Copenhagen, Denmark; 2 Department of Biology, Faculty of Science, University of Copenhagen, Copenhagen, Denmark

**Keywords:** Microbiome, Biofilms

## Abstract

Drought severely restricts plant production and global warming is further increasing drought stress for crops. Much information reveals the ability of individual microbes affecting plant stress tolerance. However, the effects of emergent bacterial community properties on plant drought tolerance remain largely unexplored. Here, we inoculated *Arabidopsis* plants in vivo with a four-species bacterial consortium (*Stenotrophomonas rhizophila*, *Xanthomonas retroflexus*, *Microbacterium oxydans*, and *Paenibacillus amylolyticus*, termed as SPMX), which is able to synergistically produce more biofilm biomass together than the sum of the four single-strain cultures, to investigate its effects on plant performance and rhizo-microbiota during drought. We found that SPMX remarkably improved *Arabidopsis* survival post 21-day drought whereas no drought-tolerant effect was observed when subjected to the individual strains, revealing emergent properties of the SPMX consortium as the underlying cause of the induced drought tolerance. The enhanced drought tolerance was associated with sustained chlorophyll content and endogenous abscisic acid (ABA) signaling. Furthermore, our data showed that the addition of SPMX helped to stabilize the diversity and structure of root-associated microbiomes, which potentially benefits plant health under drought. These SPMX-induced changes jointly confer an increased drought tolerance to plants. Our work may inform future efforts to engineer the emergent bacterial community properties to improve plant tolerance to drought.

## Introduction

Due to the intricate natural environments, plants are faced with unfavorable conditions multiple times during their growth^1^. Drought is the most common environmental stress dramatically limiting plant growth and production in agriculture^2^. Climate change and global warming is accelerating the recurrence of serious drought events, causing severe ecological and food security issues^3–5^. Considering that such events are likely to further increase, there is an urgent need for the development of sustainable solutions to improve plant resistance against drought, such as the application of beneficial microbes^6^.

Plants benefit from a wide variety of soil microbes^7^. Root-associated microbiomes play an important role in determining plant health and performance under various environmental conditions^8,9^, and the composition of root microbiome is affected by hosts and environmental factors^10,11^. Drought is one of the common environmental stresses, having significant effects on the soil microbiomes^12,13^. In addition to osmotic stress, drought often causes a strong impact on microbial composition due to increased soil heterogeneity, limited nutrient mobility and utility which aggravates plant stresses^14^. Some studies have found that certain specialized microbiomes might alleviate plant drought stress^15–17^. In turn, plant physiology and metabolism in response to drought stress can also alter the composition and structure of the microbiome with potential consequences for host adaptation and fitness^16,18^. Structural adaptations within bacterial communities in the root microbiome to abiotic and biotic stressors may provide plants with the potential to improve tolerance against drought, and further promote plant health^19–21^.

Beneficial bacteria isolated from plant roots have been identified as plant growth-promoting rhizobacteria (PGPR)^22^, as they can directly and indirectly improve plant growth and performance under stress via promoting nitrogen fixation, increasing nutrient uptake, improving soil properties, inhibiting plant pathogens and enhance plant tolerance to drought^17,23^. For example, an early study found that *Paenibacillus polymyxa* increased drought tolerance in *Arabidopsis thaliana* by regulating the expression of gene *ERD15* involved in the drought-stress response^24^. Although PGPRs have been widely studied in the past decade, most of these efforts so far have mainly focused on traits of single strain^25–27^, and are overlooking potential emergent properties of microbial communities on plant growth. Emergent properties of plant microbiota can be achieved when the bacterial community displays effects that are not observed from any of the community members when studied in isolation^28^. These emergent properties may result from the synergistic interactions among different species in the microbial community. As a result, it is possible that the functional capacity of the bacterial community or consortia is far beyond the sum of each individual due to beneficial interactions with each other^29^.

Bacteria often live as biofilms and function as communities^30,31^. Multispecies biofilm is the naturally-occurring and dominant lifestyle of bacteria in nature and their interspecies interactions can lead to mutualistic relationships or competitive activities^32–34^. In our previous studies, we had observed strong synergistic effects of four soil-isolated bacterial strains on biofilm formation as these four strains significantly produced more biofilm when co-cultured together than the sum of four mono-species biofilms^35^. Later, a recent study further found that a three-species combination among four species, composed of *Xanthomonas*, *Stenotrophomonas*, and *Microbacterium* spp., which also showed increased biofilm production compared to their individual members, induced systemic resistance (ISR) in *Arabidopsis thaliana* against phytopathogens^20^. However, whether such synergistic effects of multispecies biofilms result in emergent properties on plant drought tolerance remains largely unexplored. It is known that the main component in biofilm is water (up to 97%), which has the potential to retain water for plants during drought^36,37^. Therefore, we selected this four-species consortium to investigate its potential impact on plant drought tolerance. We hypothesized that this four-species bacterial consortium would better protect plants from drought stress compared to single species, as these four strains together have the strongest synergy on biofilm formation and significantly produce more biofilm than the sum of their four single-species biofilms^35^.

In this study, the model plant *Arabidopsis thaliana*, grown in vivo, was inoculated with four-species consortium (SPMX) composed of *Stenotrophomonas rhizophila*, *Paenibacillus amylolyticus*, *Microbacterium oxydans*, and *Xanthomonas retroflexus* to evaluate this consortium impact on plant drought tolerance. We tested more than 1200 *Arabidopsis* plants and showed that the SPMX inoculated together significantly improved plant survival under drought while no drought-tolerant effect was observed with single-strain inocula, indicating that the enhanced drought tolerance results from emergent properties of the four-species consortium rather than individual strains. Furthermore, we investigated SPMX-induced differences in plant physiology, drought-related gene expression and root-associated microbiomes, which might jointly help alleviate the negative effects of drought on plant performance. Understanding such emergent bacterial community properties may provide new opportunities to improve plant health and performance in the face of drought.

## Results

### The bacterial consortium SPMX significantly improved drought tolerance in *Arabidopsis* plants

**Fig. 1:**
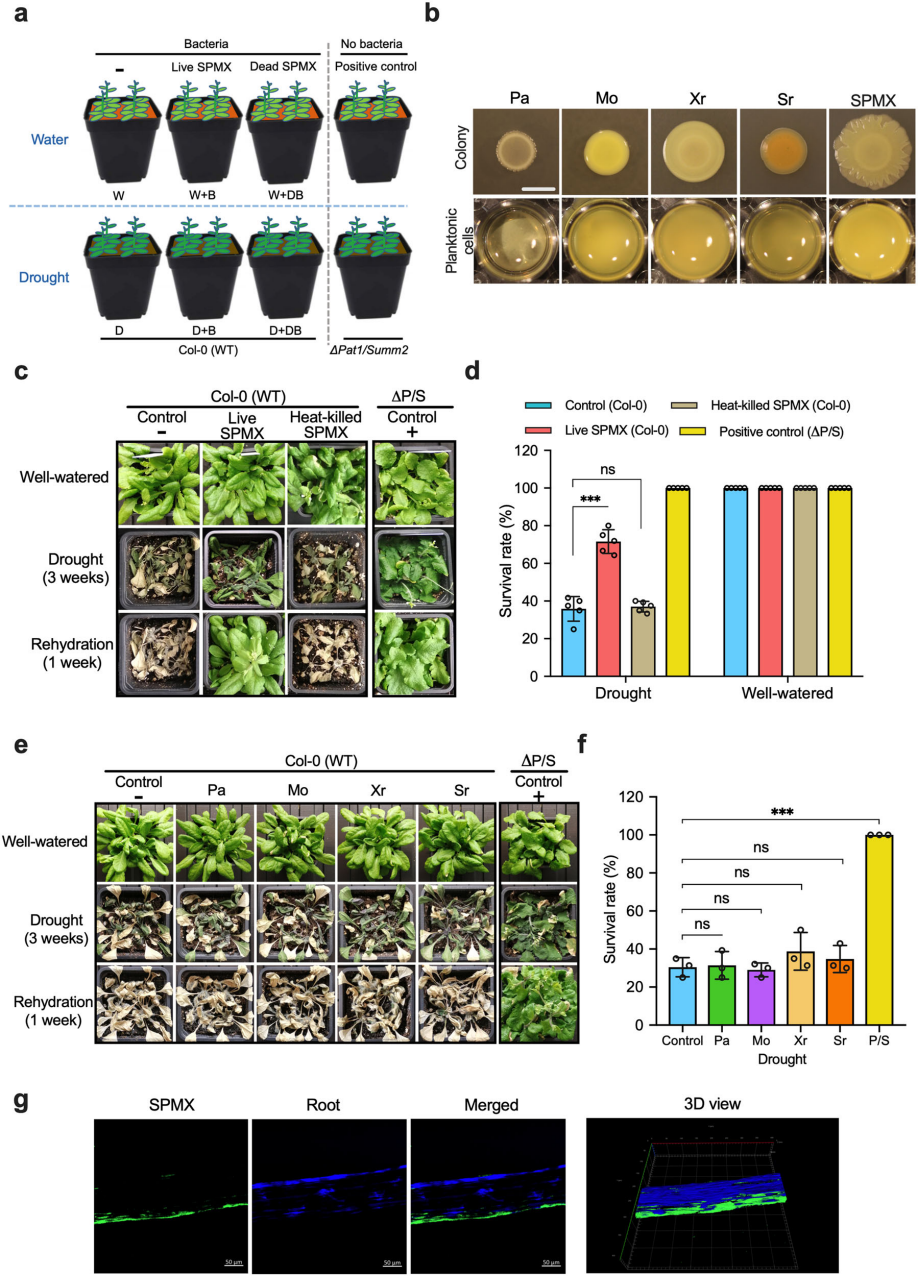
The effects of SPMX and its individuals on the survival of *Arabidopsis* plants grown under drought stress. **a** Experimental setup for plants inoculated with either living or heat-killed SPMX under well-watered and drought conditions, and corresponding short terms below the treatments. **b** Visualization of colony morphology and planktonic cells in microtiter wells of mono-species and multi-species cultures at 24 °C after 4 days and 48 h, respectively. (The scale bar = 10 mm) **c** Representative pictures of plants inoculated with live/dead SPMX under two conditions after 21 dpi and 7-day rehydration. Col-0 (WT) and drought-resistant mutant P/S plants were treated with equal volumes of saline solution as negative control (−) and positive control (+), respectively. **d** Survival rates of plants inoculated with live/dead SPMX after 21 dpi (*n* = 5 independently biological replicates, plants = 660 in total). **e** Representative pictures of plants inoculated with the four individuals under two conditions at 21 dpi drought and 7 dpr rehydration. **f** Survival rates of plants inoculated with four mono-species cultures after 21 dpi drought (*n* = 3 independently biological replicates, plants = 600 in total). **g** SPMX root colonization and biofilm formation examined by confocal microscopy. The green fluorescence along the sides of roots indicates the SPMX multispecies biofilm visualized by staining with SYTO9. The blue fluorescence visualizes the root staining with calcofluor white (CFW) (The scale bar = 50 μm). Error bars shown in **d** and **f** represent mean ± standard deviation. Asterisks indicate statistically significant differences between two survival percentage values (treated group vs. control) under drought as evaluated by one-way ANOVA followed by a Tukey HSD test: ***P < 0.001. (ns = no statistical difference).

Inspired by strong synergistic interactions on biofilm formation when Sr, Pa, Mo, and Xr co-cultured all four, compared to any other single-species, dual-species, and three-species combinations^35^ (Fig. 1b and Supplementary Fig. 1), we were, in this study, interested in whether a co-cultured SPMX consortium would also help plants to tolerate drought stress. Firstly, we investigated the effects of SPMX consortium on pot-grown *Arabidopsis* plants under 21-day drought. The experimental setup and timeline are shown in Figs. 1a and 6. Living and heat-killed SPMX suspensions were inoculated to 3-week-old *Arabidopsis* Col-0, respectively. Col-0 and drought-tolerant P/S mutants given a saline solution in equal volumes were used as negative and positive controls respectively. After withholding watering for 21 dpi drought, the leaves of Col-0 treated with living SPMX and P/S plants displayed much darker green and exhibited lighter wilt symptoms than those of negative control plants (Fig. 1c). However, none of the four strains individually inoculated onto the plants alleviated drought symptoms (Fig. 1e).

To further evaluate plant drought tolerance, we rehydrated all plants exposed to 21 dpi drought and registered the survival rate where plants survived following 21 dpi drought stress. At 7 dpr, we observed that 71.6 ± 6.3% (mean ± SD) plants inoculated with live SPMX survived and recovered from drought symptoms, while those non-SPMX inoculated (control) and heat-killed SPMX-inoculated plants only had around 35% (35.8 ± 6.6% and 36.9 ± 3.3%, respectively) recovery rate (Fig. 1c, d). No significant difference was observed between control and single-species inoculated plants (Fig. 1e, f). Drought-tolerant mutant P/S, as a positive control, 100% survived 21 dpi drought (Fig. 1d, f). The increased viability at 21 dpi drought suggested the emergent property of living SPMX consortium in plant drought tolerance which each species did not display. Under well-watered conditions, all treated plants and control plants had a survival rate of 100%. To investigate the ability of SPMX to form biofilm on plant roots, five-day-old *Arabidopsis* plants grown on MS medium were root-inoculated with SPMX multi-culture. After 4-day co-cultivation with SPMX, biofilm formation was observed on the root surface by confocal microscopy (Fig. 1g), which indicated that SPMX was capable of colonizing the plant root and subsequently formed biofilm.

### SPMX-induced drought tolerance promoted plant growth under drought

**Fig. 2:**
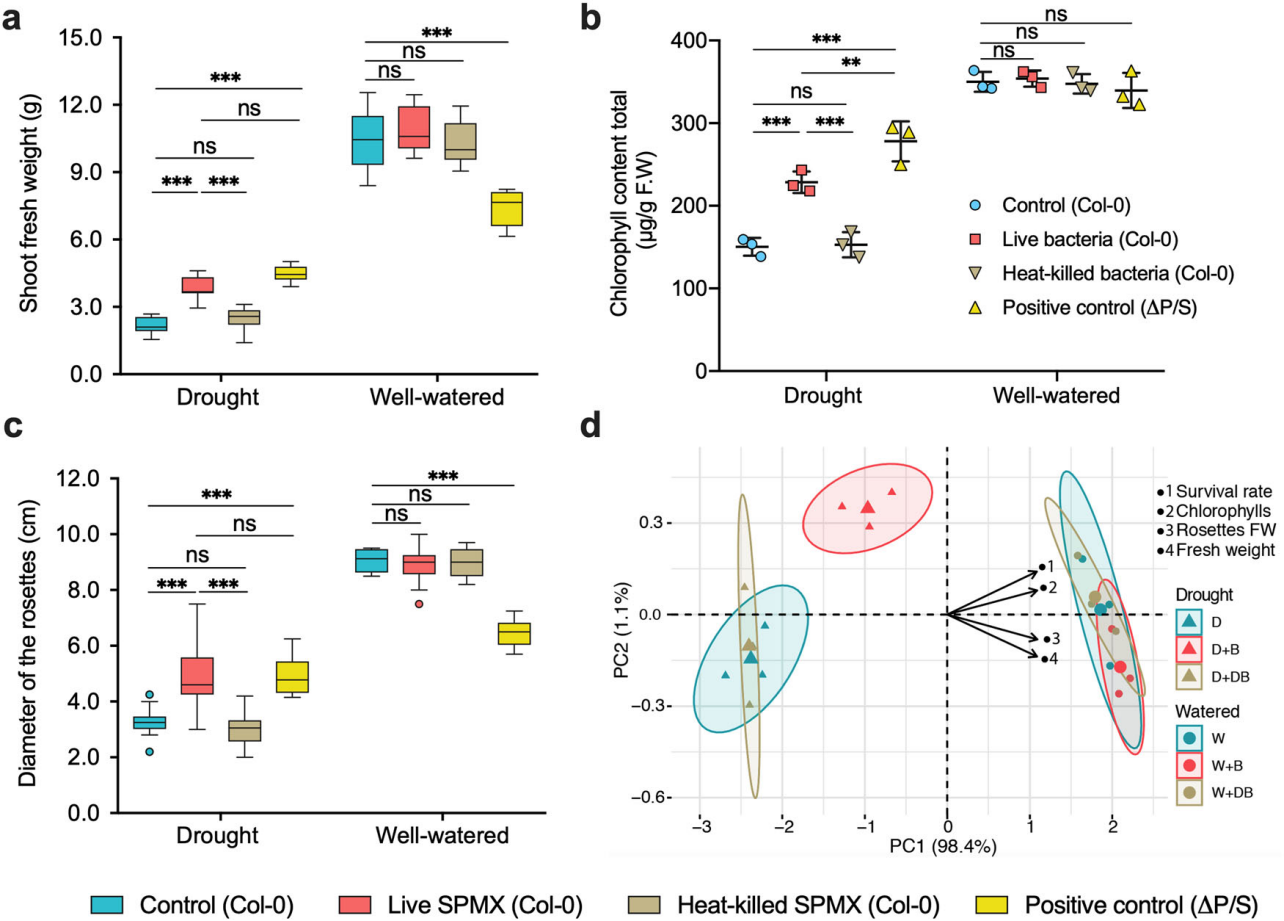
The effect of inoculation with either living or heat-killed SPMX on plant growth. The physiological differences were shown in **a** shoot fresh weight (g) (*n* = 9, nine replicates from three independent experiments); **b** total chlorophyll content (μg/g) (*n* = 3, three independent experiments) and **c** rosette diameter (cm) under fresh weight (FW) after 21 dpi of drought or watered treatment (*n* = 10 replicates from three independent experiments). **d** Biplot display of PCA of the parameters analyzed in *A. thaliana* Col-0 pot-grown plants. Treatments: W (well-watered condition) and D (drought condition), inoculated with SPMX (W + B) and (D + B), and with dead SPMX (W + DB) and (D + DB), respectively; variables: survival rate (%), rosette diameter FW (cm), fresh weight (g) and chlorophyll content (μg/g FW). Error bars represent mean ± standard deviation in **a** and **b**. Asterisks above histograms indicate whether two group percentage (different treatment vs. control) under drought are statistically significantly different as assessed by one-way ANOVA followed by a Tukey HSD test: **P < 0.01, ***P < 0.001. (ns = no statistical difference).

To evaluate the impacts of SPMX on plant drought tolerance, we determined the plant growth under both drought and well-watered conditions at 21 dpi. Plants exposed to drought had notable decreases in fresh weight and diameter of rosettes compared to well-watered plants. Consistent with drought-tolerant Δ*P*/*S*, the fresh weight (g) and diameter of rosettes (cm) of those living SPMX-inoculated plants were significantly increased by approx. 2-fold and 1.5-fold, respectively, compared to non-SPMX and dead SPMX-treated controls under drought (4.0–2.0 g, 5.0–3.5 cm) (Fig. 2a, c). Due to the innate phenotype difference between mutant Δ*P*/*S* and wild type Col-0, the fresh weight and diameter of rosettes of Δ*P*/*S* plants were significantly lower than that in non-SPMX, living SPMX and dead SPMX treated Col-0 plants under well-watered conditions (Fig. 2a, c).

Similarly, the content of leaf chlorophylls *a*+*b* at 21 dpi was highest in drought-tolerant Δ*P*/*S* when exposed to drought, followed by living SPMX-inoculated Col-0 that was 1.5-fold higher than those in two controls (non- and dead SPMX-treated Col-0) (Fig. 2b). This indicates that SPMX helped to sustain chlorophyll content in plant leaves during drought, which might contribute to photosynthetic efficiency. No significant difference in chlorophyll contents was observed between Col-0 and Δ*P*/*S* plants under well-watered conditions (Fig. 2b). Furthermore, none of the individual strains was able to significantly affect plant growth compared with control under neither drought nor well-watered conditions (Supplementary Fig. 2), which again indicated that this enhanced drought tolerance resulted from the emergent properties of co-cultured SPMX rather than its individual members.

Figure 2d shows the biplot graph for 6-week-old *Arabidopsis* Col-0 plants grown in pots both with drought (D) and watering (W) treatment. The matrix for the principal component analysis (PCA) consisted of six cases corresponding to the combination of the two irrigation conditions (W and D) and inoculant treatments (non-inoculated and SPMX-inoculated live: B/dead: DB), and four variables (Fig. 1a). Obviously, SPMX-treated plants under drought (D + B) could be separated from the drought treatments (D and D + DB), and were close to the three groups under well-watered conditions. Besides, the plant survival rate was positively associated with SPMX treatment (D + B). Combined, these results further confirm the positive effects of SPMX on plant drought tolerance.

### Plant endogenous abscisic acid (ABA) signaling was affected by addition of SPMX under drought

The plant phytohormone ABA plays a critical role in the response to drought stress and quickly accumulates when the plant is exposed to dehydration^38–40^. ABA induces stomatal closure and prevents transpiration-caused water loss and thereby confers increased drought tolerance to the plant^41,42^. To confirm this enhanced drought tolerance, we performed quantitative RT-PCR (qRT-PCR) to examine expressions of four ABA-related marker genes, ABA biosynthesis gene *NCED3* (*9-cis-epoxycarotenoid dioxygenase 3*), ABA responsive gene *COR15A* and two drought-responsive genes *RAB18* and *TSPO*^43–47^ (Supplementary Table 1), in plants inoculated with or without SPMX (control) when exposed to drought at 0, 2, 7, 14 dpi.

**Fig. 3:**
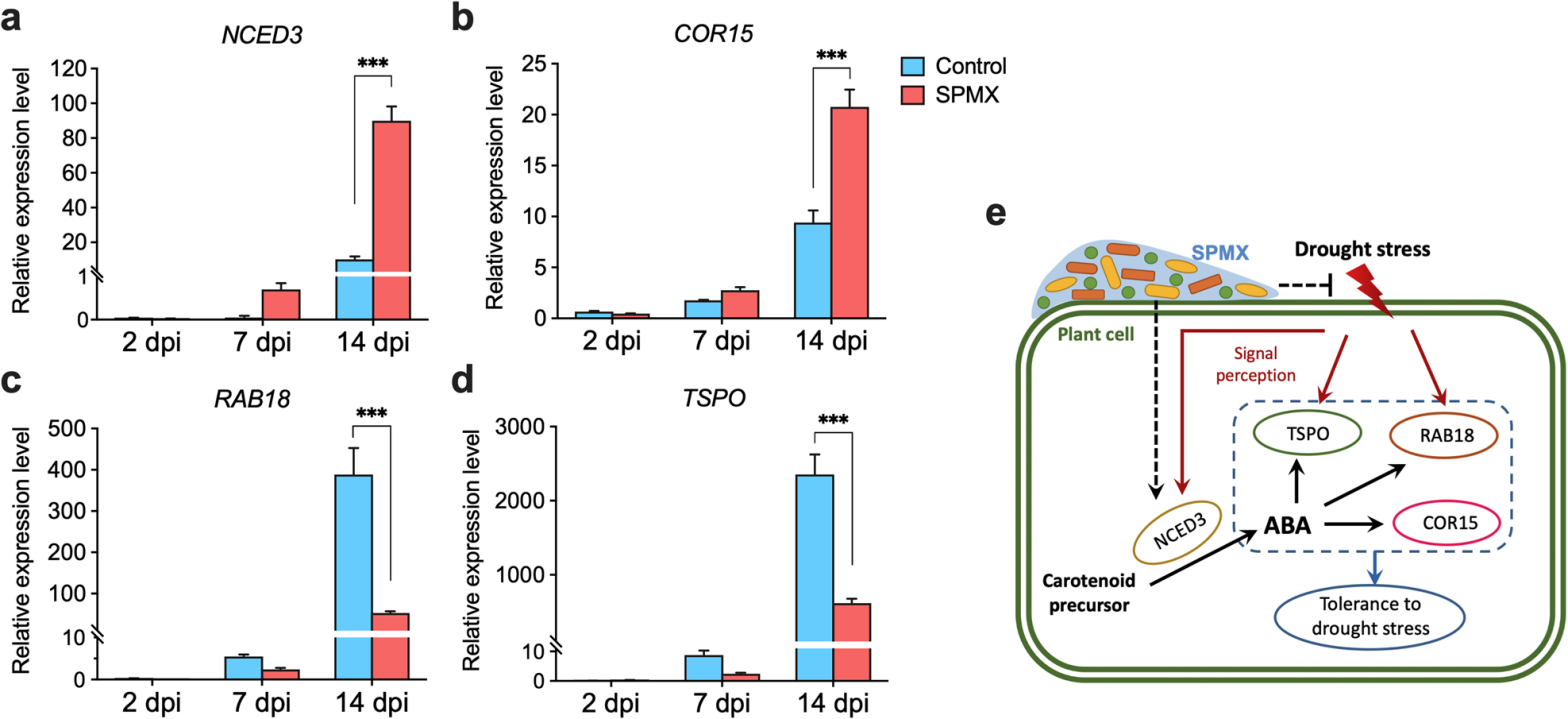
The relative expression levels of the ABA pathway-related genes under drought when inoculated with SPMX. Differential expression in **a** ABA-biosynthetic gene *NCED3*; **b** ABA-responsive gene *COR15A*; **c** drought-responsive gene *RAB18*; **d** drought-responsive gene *TSPO* in plants inoculated with and without SPMX (control) at 2, 7, and 14 dpi (relative to the expression level of corresponding genes at 0 dpi). *ACT2* was used as an internal control. Error bars represent mean ± standard deviation (three replicates, *n* = 3). At least two independent replicates showed similar results, with one shown here. **e** A model suggesting the potential effects of SPMX on ABA-related signaling pathways in response to drought. The regulations of four maker genes *NCED3*, *COR15*, *RAB18*, and *TSPO* involved in ABA-biosynthetic and ABA-responsive pathways in response to drought, which have been already reported, are shown with solid arrows, while potential influences of SPMX on ABA signaling proposed in the present study are depicted by broken lines. Arrow heads and end lines indicate positive and negative regulation, respectively. Asterisks indicate statistically significant difference assessed by ANOVA followed by a Tukey HSD test: ***P < 0.001.

The key ABA biosynthesis enzyme *NCED3*^48^ was strikingly induced (up to 5-fold) by SPMX inoculation under drought especially at 14 dpi (stopped watering for 14 days) (Fig. 3a) compared to that in non-SPMX controls, indicating that the addition of SPMX might promote ABA biosynthesis during drought. Consistently, the ABA-responsive gene *COR15A* was significantly upregulated in SPMX-inoculated plants compared to the controls at both 7 dpi and 14 dpi under drought probably due to increased ABA biosynthesis, which also reflect an enhanced response to ABA in SPMX-inoculated plants (Fig. 3b). However, the two stress-responsive genes *RAB18* and *TSPO* were dramatically down-regulated in SPMX-inoculated plants at both 7 dpi and 14 dpi (Fig. 3c, d) compared to the controls, which might reflect a reduced drought stress sensed by plants inoculated with SPMX. Collectively, the differential expression of these four marker genes between SPMX-inoculated plants and non-SPMX controls under drought indicated that the addition of SPMX affected endogenous abscisic acid (ABA) signaling, which might reflect an increased plant drought tolerance and reduced drought stress when inoculated with SPMX consortium under drought (Fig. 3e).

### The relative abundance of SPMX was higher in rhizoplane than in rhizosphere

**Fig. 4:**
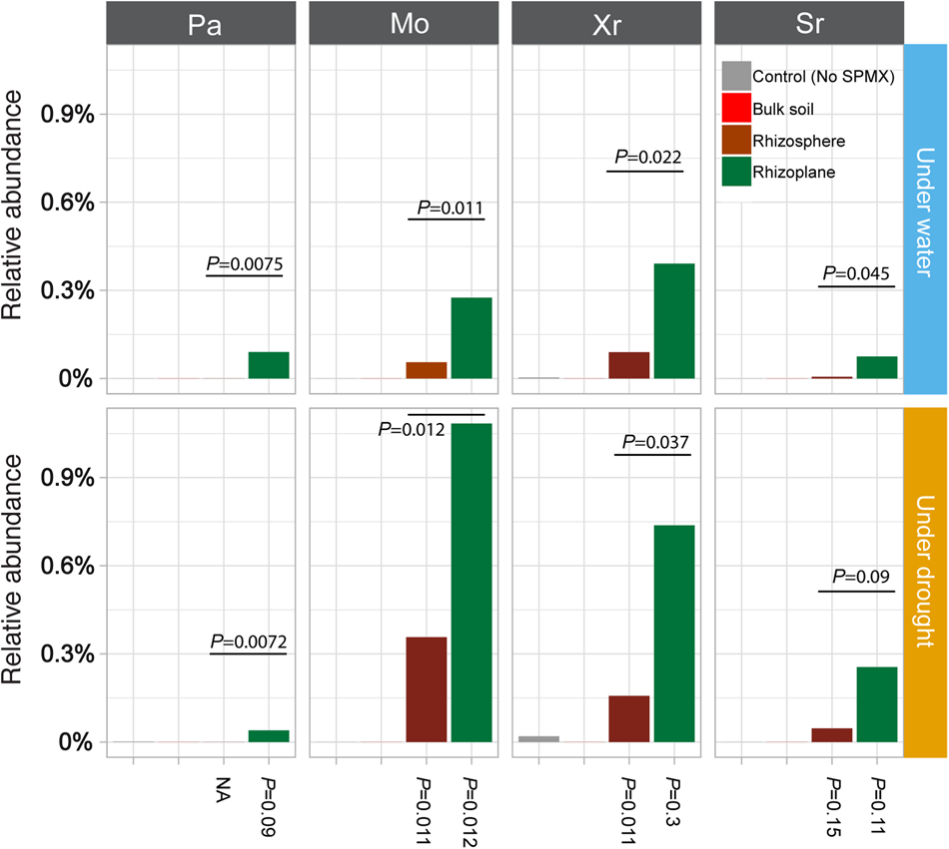
The relative abundance of four individual strains in the root-associated microbiome. The relative abundance of each strain colonized in the bulk soil (red), rhizosphere (dark red) and rhizoplane (dark green) of SPMX-inoculated plants at 21 dpi under either regular water conditions (upper bar chart), or drought conditions (lower bar chart). The relative abundance of each strain in the control (gray) presents a sum of their abundance in the rhizosphere and rhizoplane of non-SPMX inoculated control plants, respectively. FDR adjusted *P* values generated by Wilcoxon rank-sum test presented above and below bar chart indicate statistical significance in relative abundance of individual strains between rhizosphere and rhizoplane (above), and between under water and drought conditions (below), respectively. Data based on five samples (*n* = 5) collected in each treated group.

The rhizosphere is the narrow zone between the root surface and the soil, which is directly influenced by root secretions and where microorganisms interact strongly with plant roots whereas the rhizoplane is the layer of bacterial cells directly in contact with the plant root^8,49^. To study the root colonization of SPMX, we quantified and analyzed the relative abundance of each strain of SPMX colonizing the rhizosphere and rhizoplane at 21 dpi by mapping full-length *16S* rRNA gene sequence of SPMX against ASVs obtained in this study. The combined relative abundance of each strains from the rhizosphere and rhizoplane of plants grown with no SPMX inoculation were used as control. Only a very low relative abundance of Xr (0.0019% under drought and 0.0036% well-watered conditions) and Pa (0.0014% under drought) were observed in these control samples (Fig. 4).

In samples inoculated with SPMX we found, under well-watered conditions, that Xr was the most abundant among the four strains in the rhizoplane, at 0.40%, followed by Mo (0.27%), Pa (0.09%), and Sr which is the lowest below 0.08% (Fig. 4). All of these four relative abundances for Pa, Mo, Xr, and Sr in the rhizoplane were significantly higher than those in the rhizosphere (all FDR adjusted *P* < 0.05). However, under drought, the relative abundance of Mo was the highest among SPMX consortium both in the rhizosphere and rhizoplane, attaining 1.05%, while abundances of Xr, Sr, and Pa were lower at 0.75%, 0.25%, and 0.04% in the rhizoplane, respectively (Fig. 4). These relative abundances for SPMX on the rhizoplane were also significantly higher than those in the rhizosphere (FDR adjusted *P* < 0.05) except for Sr (FDR adjusted *P* = 0.09). No ASVs belonging to SPMX were found in the corresponding bulk soil (Fig. 4). Basically, the relative abundance of SPMX colonizing the rhizoplane was notably higher than those colonizing the rhizosphere under both conditions (Fig. 4), which might suggest that SPMX enhanced colonization ratio in the rhizoplane compared to that in the rhizosphere. Moreover, the relative abundance of Mo was significantly enhanced in the both rhizosphere and rhizoplane under drought conditions compared to that under well-watered conditions (FDR adjusted *P* = 0.011 and 0.012, respectively).

### SPMX affects root-associated microbiome in *Arabidopsis* under drought conferring the potential of increased drought tolerance to plants

To investigate the impact of SPMX on root microbiome under drought, four groups of root-associated soil (W: under well-watered conditions; W + B: SPMX-inoculated under W; D: under drought conditions and D + B: SPMX-inoculated under D) from rhizosphere, rhizoplane, and bulk soil samples were collected at the endpoint of 21 dpi before re-watering (Fig. 1a), and microbial alpha-diversity and beta-diversity were analyzed. Different soil compartments (rhizosphere/rhizoplane/bulk soil) explained the main variation in microbial compositions (Supplementary Fig. 3), the bulk, rhizosphere and rhizoplane soil samples all had significantly different microbial compositions compared one to another (FDR adjusted *P* = 0.001, 0.001, and 0.001, respectively).

**Fig. 5:**
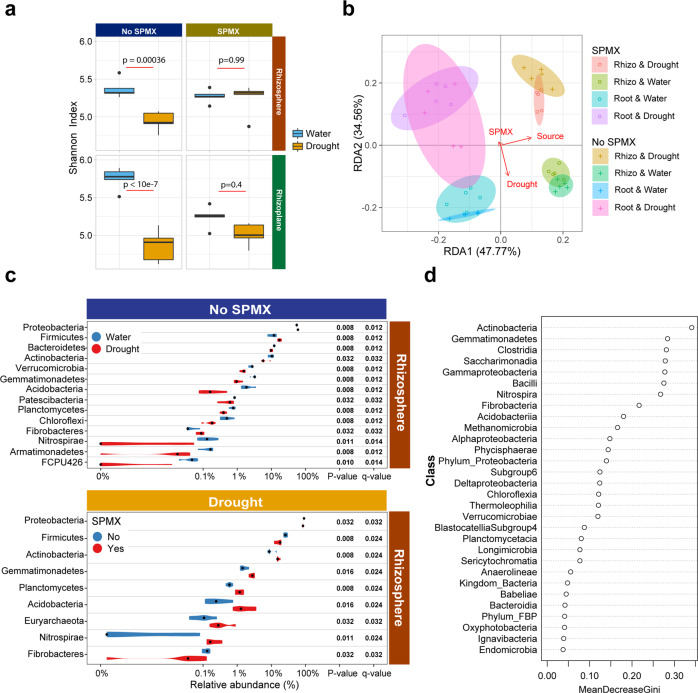
The influences of drought stress and SPMX on root microbial diversity and composition. **a** Boxplots of Shannon’s Diversity in drought (yellow) and water (blue) for two soil compartments (rhizosphere/rhizoplane) with and without SPMX inoculation. *P* > 0.05 indicates that drought treatment groups were not significantly different from the respective watering control group by one-way analysis of variance (ANOVA). **b** Redundancy analysis (RDA) plot for all soil samples generated using the Bray–Curtis distance; samples are colored for each combination of soil compartments (rhizosphere/rhizoplane), inoculation treatment (SPMX/non-SPMX) and water treatment (drought/water). Control = No SPMX, Rhizo = Rhizosphere, Root = Rhizoplane. **c** All the significantly differential bacterial phyla at RA in the rhizosphere between drought (red) and well-watered (blue) conditions without SPMX inoculation (upper chart); All the significantly differential bacterial phyla in the rhizosphere when inoculated with SPMX (red), compare to that inoculated with no SPMX (blue) under drought; Only statistically significant values (FDR adjusted *P* < 0.05, Wilcoxon rank-sum test) were shown. **d** The importance of taxa at class level under drought by random forest. Mean decrease in Gini was used to evaluate the importance level of bacterial classes affected by SPMX in the rhizosphere under drought. Data based on five samples (*n* = 5) collected in each treated group.

Drought stress significantly decreased the microbial alpha diversity of bacterial communities in two soil compartments (rhizosphere/rhizoplane) in the non-SPMX control (Fig. 5a), consistent with recent studies^12^. However, in the presence of SPMX, drought did not significantly lower the microbial diversity in both of the two root-associated communities, although the mean of diversity in the rhizoplane under drought was slightly lower than that under well-watered conditions (Fig. 5a). This suggested that SPMX might protect root-associated microbial diversity from drought.

Next, we performed RDA analysis and quantified the influence of three variables including SPMX, drought and soil compartments on microbial diversity through partition of sum of variations. As shown in the RDA plot (Fig. 5b), the microbial beta diversity with the eight combinations of conditions differed significantly between each other (Pairwise PERMANOVA, FDR adjusted *P* = 0.001 for all the comparisons). Besides, SPMX, drought and soil compartments shown in the RDA plot significantly influenced microbiome compositions (Permutation test, FDR adjusted *P* = 0.001, 0.001, and 0.001, respectively) and had successively larger effects (adjusted *R*2 = 2.1%, 6.85%, and 10%, respectively) (Supplementary Fig. 4) on microbial diversity. This indicated that SPMX re-shaped the root microbiomes under the drought conditions.

We further investigated the differential bacterial phyla at relative abundance (RA) between soil samples inoculated with and without SPMX under drought or well-watered conditions. Firstly, concurring with previous studies, phyla Proteobacteria, Firmicutes, Actinobacteria, Bacteroidetes, Acidobacteria, and Planctomycetes constituted the core root microbiome in *Arabidopsis*^10,50^. Proteobacteria (over 50%) was the most abundant phylum, followed by Firmicutes, Bacteroidetes and Actinobacteria, and they accounted for 80% abundance of the whole microbiome (Supplementary Fig. 5). We next performed statistical analysis for all differential bacteria significantly responding to drought stress and SPMX at phylum level on RAs. In absence of SPMX, drought stress significantly influenced 9 phylum RAs in the top ten most abundant phyla in the *Arabidopsis* rhizosphere (Fig. 5c). Consistent with previous studies^14,51^, two dominant phyla Proteobacteria (59%) and Firmicutes (17%), that are normally enriched in moisture-limited soils, were also significantly enhanced under drought in this study. In contrast, RAs of other seven major phyla, Bacterioidetes, Actinobacteria, Verrucomicrobia, Gemmatimonadetes, Acidobacteria, Patescibacteria, and Planctomycetes were notably decreased in response to drought (Fig. 5c). Among them, Bacteroidetes and Planctomycetes were also typically drought-depleted in most drought cases^14,52,53^. Similar phylum changes were observed in the rhizoplane (Supplementary Fig. 6). However, under drought when inoculated with SPMX, the RAs of both drought-enriched Proteobacteria and Firmicutes significantly decreased, whereas drought-depleted Actinobacteria, Gemmatimonadetes, Planctomycetes, and Acidobacteria in rhizosphere (Fig. 5c), and Bacteroidetes in rhizoplane (Supplementary Fig. 7) were all enriched.

We further investigated all classes presenting statistically significant differences under drought. Among classes (RA > 10%), Actinobacteria (2.1-fold change) and Alphaproteobacteria (1.2-fold change) significantly rose in the presence of SPMX (Supplementary Figs. 8 and 9). Furthermore, random forest analysis also predicted the importance of the Actinobacteria when inoculated with SPMX (Fig. 5d). These results suggested that the Actinobacteria might be most influenced by SPMX addition during drought. Taken together, SPMX-mediated stability in root microbial diversity and root microbiome shifts during drought may jointly alleviate drought stress and confer the drought-tolerant potential to plants.

## Discussion

Our data demonstrated that an addition of a four-species bacterial consortium (SPMX) could help improve plant performance and survival under drought. This induced drought tolerance was observed when plants were inoculated with four-strain SPMX together, while no drought tolerance was induced when each strain was inoculated individually, indicating that this enhanced drought tolerance resulted from emergent properties of the SPMX consortium. (Figs. 1 and 2). While the rhizobiome has previously been considered as an extended root phenotype^54^ and several studies report the implication of root-associated microorganisms in drought tolerance^14,55^. This is, to our knowledge, the first demonstration of a minimal bacterial community emergent property leading to drought stress protection in a plant host.

We therefore moved on to investigate the potential mechanisms underlying the SPMX-induced drought tolerance. Biofilm formed by SPMX may be partly responsible for this increased drought survival due to their known ability to produce high levels of hydrated polymers in the matrix to retain water^56,57^. Our confocal microscopy analysis revealed SPMX ability to form biofilm on root surface (Fig. 1g). Recent studies also indicated the potential of biofilm in drought stress alleviation^15,58^. Besides, photosynthesis is the essential way for plants to obtain energy and its efficiency associates positively with chlorophyll contents^59^. In this study, the addition of SPMX increased chlorophyll contents of *Arabidopsis* under drought (Fig. 2), which would be helpful to maintain plant growth under drought due to potentially enhanced photosynthesis. ABA has been widely reported as a central regulator regulating the plant responses to drought stress via closing stomata to prevent water loss and inducing related genes to enhance drought tolerance^38,42^. Differences in expression levels of four ABA-related marker genes under drought suggested that the addition of SPMX affected the ABA signaling under drought. Upregulated ABA-biosynthetic gene *NCED3* and ABA-responsive *COR15* by SPMX addition (Fig. 3a, b) reflected a possible enhanced ABA biosynthesis, which is known to increase plant drought tolerance^60^. ABA-responsive genes such as *RAB18*, *TSPO*, and *RD29B* can be also induced by drought stress^61–63^ (Fig. 3e). Some studies have shown that *RAB18* and *TSPO* were upregulated by environmental water-limited stress^43–45^. However, in our study, the expression of drought-responsive *RAB18* and *TSPO* were significantly downregulated. The reduced expression of these two genes might reflect a decreased drought stress sensed by plants. This is probably due to the ability of the biofilm to retain water for plants, thereby reducing the water stress sensed by plants under drought. Combined, expression changes in these four maker genes suggested a possibility of increased drought tolerance and reduced drought stress when plants were inoculated with the SPMX consortium under drought.

To study the root colonization of SPMX, we evaluated the relative abundance of each species in SPMX established in two rhizo-compartments via amplicon sequencing. Under watered conditions, Xr was the most abundant species in both rhizosphere and rhizoplane (Fig. 4). Intriguingly, we found that Mo became the most abundant rather than Xr during drought both in the rhizosphere and rhizoplane, while in our previous studies, Mo was always at the lowest ratio in SPMX-formed biofilm when no stress factor was active^64^. It suggested that Mo may play an important role under drought. It is worth-noting that the relative abundance of each SPMX strain was enhanced in the rhizoplane compared to that in the rhizosphere (Fig. 4), which may indicate an enhanced colonization ratio of SPMX in the rhizoplane compared to in the rhizosphere. It also surprised us that the low abundant SPMX could have such a significant impact on plant drought tolerance, which leads us to speculate whether the observed effects might also include an indirect effect derived from the addition of SPMX to the soil. Therefore, we further investigated possible rhizo-microbiome shifts caused by SPMX that may benefit plants against drought.

Drought stress is considered as a negative abiotic factor that reduces microbial variety and abundance in the soil^12,14,65^. Our data further indicated that drought had a larger impact on microbiome structure in rhizosphere compared to that in rhizoplane due to more core phyla affected and changed in rhizosphere (Fig. 5c and Supplementary Fig. 6) (9 phyla in rhizosphere vs. 4 in rhizoplane). Similarly, under drought, 9 phyla in rhizosphere significantly responded to SPMX addition compared to those in rhizoplane (2 phyla) (Fig. 5c and Supplementary Fig. 7). In particular, our results showed that the SPMX stabilized the diversity of the root microbiome during drought (Fig. 5a), which potentially benefited plant growth under drought^14^. Furthermore, SPMX significantly reshaped the root microbiomes during the drought (Fig. 5b, c and Supplementary Fig. 4). Interestingly, we found that these drought-impacted, SPMX-reshaped microbiomes were similar to the bacterial composition under watered conditions. This was reflected by the remarkable rise of the five drought-depleted phyla and drop of two dominant drought-enriched phyla when added SPMX under drought (Fig. 5c and Supplementary Fig. 7). Therefore, these RA-reversed phyla in response to SPMX addition under drought may further indicate a reduced water stress, as also reflected in the observed downregulation of the stress-responsive genes *RAB18* and *TSPO* (Fig. 3c, e), which likely benefits from water retained by the biofilm formed in the SPMX-inoculated condition. More work will be needed to confirm if this SPMX-formed biofilm observed here in vitro is also able to be formed in the soil.

Alternatively, certain beneficial microbes are specifically recruited or enriched in presence of SPMX to help plants deal with drought stress. As we analyzed, Actinobacteria might be most influenced by the addition of SPMX under drought and may benefit plant tolerance to drought. Many of the known Actinobacteria strains were identified as PGPR to improve the plant’s multi-stressed tolerance and seedling vigor in water-restricted soil^66,67^. Furthermore, the enrichment for Actinobacteria during drought was not a random event but was most likely an intrinsic and natural microbial adaptation in response to drought^12^. The emergent properties of SPMX may strengthen such potentially mutually-beneficial relationships, although further evidence will be required to test this hypothesis.

## Methods

### Plant materials and growth conditions

*Arabidopsis thaliana* ecotype Columbia (Col-0) was used for drought experiments in this study. The double mutant of At1g79090 (PAT1) *pat1* (Salk_040660) and At1g12280 (SUMM2) *summ2*-8 (SAIL_1152A06) pat1/summ2 tolerant to drought stress^68^ was used as positive control in this study. Surface-sterilized seeds were grown on solid Murashige–Skoog (MS) salts medium (Duchefa), with 1% sucrose and 0.8% agar, vernalized 48 h at 4 °C and then placed in the growth chamber with 150 μmol m^−2^ s^−1^ light intensity, 70% humidity, 12/12 h light/dark photoperiod, 22 °C for daytime temperature and 21 °C for night to allow germination. Then, 10-day-old germinated seedlings were transplanted in a 7 cm × 7 cm square pots with drainage holes (four plants in each pot), containing approximately 60 g of non-sterile soil (Plug og såjord, SW HORTO A/S, DK) (recipe described in Supplementary Table 2) to grow in the chamber with the same conditions.

### Bacterial strains and growth conditions

The inoculated four-species model consortium consists of *Stenotrophomonas rhizophila* (Sr), *Paenibacillus amylolyticus* (Pa), *Microbacterium oxydans* (Mo), and *Xanthomonas retroflexus* (Xr), termed as SPMX for short hereafter. These four strains were isolated and identified during previous studies and found to exhibit synergistic biofilm formation capabilities^35,69^. The four strains from frozen glycerol stocks were streaked on tryptic soy agar (TSA) (Sigma, St. Louis, USA). Plates were incubated at 24 °C for 48 h. Single colonies were inoculated in 5 ml TSB culture tubes, and incubated overnight with shaking with 250 rpm at 24 °C in an orbital shaker. Overnight cultures were then inoculated in 100 ml TSB and incubated under the same conditions.

### Preparation of bacterial suspension and inoculation

Bacterial cells from overnight cultures were centrifuged at 5000×*g* for 5 min to harvest cell pellets that were then washed and resuspended in 0.3% sterile saline solution (0.3% NaCl). Each cell suspension was adjusted to yield ~10^8^ CFU mL^−1^ before use based on optical density (OD_600_ = 1.0 for Mo, Xr, and Sr; OD_600_ = 2.0 for Pa) and serial dilutions with plate counts. For mixed cultures of bacterial consortium SPMX (B), equal volumes of each strain suspension were mixed in a ratio of 1:1:1:1 (v/v/v/v) for use. The mixed culture was further autoclaved in 121 °C for 20 min to obtain the heat-killed bacteria (DB) used as negative control. Three-week-old plants, grown in pots in the growth chamber, were inoculated with either 5 ml SPMX suspension or 5 ml individual strain’s suspension on the soil around each plant root (5 ml bacterial suspension was slowly added at surface of soil around roots with a 10 ml sterile syringe). Equal volumes of DB suspension and sterile saline solution were added in the same way as negative controls. *Arabidopsis* drought-resistant mutant plants *pat1/summ2* (P/S)^70^ were treated with equal amounts of sterile saline solution as positive control.

### Pot-grown experiments with drought

**Fig. 6:**
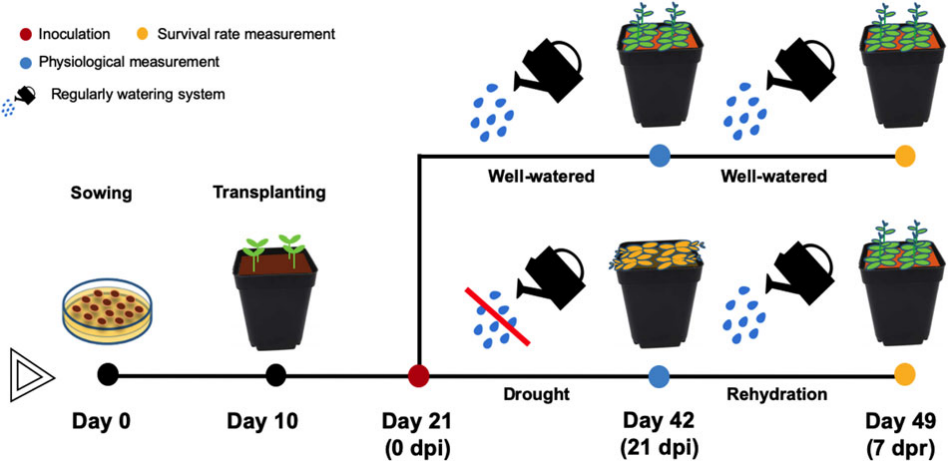
The timeline for plant experiments under drought and well-watered conditions. At day 21 (0 dpi), three-week-old *Arabidopsis* plants were inoculated with different bacterial suspension including SPMX, dead SPMX and each individual strain of SPMX. After that, for well-watered treatment, inoculated plants were watered regularly every three days (500 ml of distilled water per tray each time, 20 pots were put in each tray). Inoculated plants were treated with drought by withholding watering for 21 days. At day 42 (21 dpi), regularly watering was resumed for the inoculated plants exposed to 21-day drought. To evaluate drought tolerance, survival rate of each treatment was measured after 7 days post re-watering (7 dpr).

Three-week-old Col-0 plants were treated with inoculants and then subjected to drought treatment for 21 days of post-inoculation (dpi) by withholding water. Well-watered plants were kept under a regular watering system (W), watering every 72 h (500 ml of distilled water was poured into the tray each time, 20 pots were put in each tray, a mean of four plants grown per pot). The drought-treated plants (D) were subjected water stress by withholding water, resulting in six different treatment groups along with inoculation: (1) D; (2) D + B; (3) D + DB; (4) W; (5) W + B; (6) W + DB (Fig. 1a). The treatments and groups were arranged in a completely randomized design (20–32 plants per treatment group in each independent replicate). After 21 dpi, all plants exposed to drought were re-watered for 7 days. After 7 dpr (days post re-watering), the percentage of plant recovery in each group from drought was assessed to evaluate plant drought tolerance (Fig. 6). Plants that can be recovered from drought phenotype after 7 dpr were survived from 21 dpi drought, which was regarded as tolerance to drought, whereas those plants that cannot recover from 7 dpr re-watering were considered to be dead and not tolerant to drought. At the time point 21 dpi, physiological indicators such as the shoot fresh weight, the total chlorophyll content and the diameter of the rosettes were measured.

### Chlorophyll quantification

The leaf chlorophyll content was determined at 21 dpi in watered and drought plants inoculated with live or dead bacteria or without bacteria by following Liang’s procedure with a few modifications^71^. Plant samples were weighed and then ground in 80% acetone with sand to extract total chlorophyll. The mixture was centrifuged at 2000×*g* for 5 min to pellet any residual materials, and the absorbance of the supernatant was measured at wavelengths 645 and 663 nm. The concentration of chlorophyll *a*, chlorophyll *b*, and total chlorophyll were estimated according to the classic Arnon’s equations^72^.

### RNA extraction and reverse-transcription quantitative PCR analysis

RNA extraction and RT-qPCR analysis were performed with Zuo et al. method with a few modifications^73^. Total RNA from plant tissues were extracted with TRIzol Reagent (Invitrogen, ThermoFisher, USA), 1 μg total RNA was treated with DNAse I (Thermo Scientific) and reverse transcribed into cDNA using RevertAid First Strand cDNA Synthesis Kit according to the manufacturer’s instructions (Thermo Scientific). The constitutively expressed gene *ACT2* encoding the actin monomer was used as an internal control. qPCR was performed on a Bio-RAD CFX96 system with SYBR Green master mix (Thermo Scientific). Primers are listed in Supplementary Table 1.

### Sample collection of rhizosphere and rhizoplane

Root microbiomes were separated from two rhizocompartments: the rhizosphere (≤1 mm soil from the root surface) and the rhizoplane (on the root surface) with the method developed by Lundberg et al. and Edwards et al. with a few modifications^11,50^. Samples were collected at the end point of 21 dpi under both drought and watering conditions inoculated with or without bacteria before re-watering. The soil and plant were removed from each pot and the roots were removed from the soil. We randomly selected five pots in each treated group (*n* = 5 per treatment) to collect rhizosphere and rhizoplane samples, respectively. The excess soil was manually shaken from the roots, leaving approximately 1 mm of soil still attached to the roots. The roots with ~1 mm soil attached were placed in a sterile 15 ml Falcon tube with 5 ml of sterile phosphate buffered saline (PBS) solution, then vortexed for 30 s at maximum speed. The soil that was cleaned from the roots was stored as the rhizosphere compartment at −20 °C until DNA extraction the same day. The roots cleaned by first vortex were then picked out and placed in another new 15 ml Falcon tube with 5 ml of sterile PBS, and tightly adhering microbes at the root surface were removed using a sonication. The roots in the Falcon tube were sonicated for 3 min at 50–60 Hz (output frequency 45 kHz). The roots were then removed and discarded and the liquid PBS fraction was kept as the rhizoplane compartment.

### Rhizobacterial DNA extraction and sequencing library preparation

Genomic DNA of the treated samples was extracted using the NucleoSpin® 96 Soil DNA Isolation Kit optimized for epMotion® (Macherey-Nagel, Düren, DE) using the epMotion® 5575 robotic platform model (Eppendorf) by following the manufacturer’s protocol. Sterilized PBS solution was included during DNA extraction as blank extraction control. The hypervariable V3–V4 region of 16S rRNA gene was amplified with 2 µl template DNA, using 5 µl 5× Phusion buffer HF, 0.5 µl 10 mM dNTPs, 0.25 µl Phusion high-fidelity (HF) DNA Polymerase (Thermo Fisher Scientific, Waltham, MA, USA), 1 µl 10 µM of each primer (the modified broad primers 341F (5′-CCTAYGGGRBGCASCAG-3′) and Uni806R (5′-GGACTACNNGGGTATCTAAT-3′)^74^ in a 25 µl PCR reaction volume. The first PCR program included 30 s at 98 °C, 30 cycles of 5 s at 98 °C, 15 s at 56 °C, and 72 °C for 10 s, and then 5 min at 72 °C. Then the primers were barcoded in the second PCR with only 15 cycles. Molecular grade water and mock community were included in PCR amplification as negative and positive control, respectively. All final PCR products were purified using Agencourt AMPure XP beads (Beckman Coulter Genomics, MA, USA) with the 96-well magnet stand. The purified second PCR products were normalized by the SequalPrepTM Normalization Plate (96) kit (Invitrogen Ltd., Paisley, UK) and then pooled in equimolar concentrations. The pooled library was concentrated using the DNA Clean & Concentrator™-5 Kit (Zymo Research, Irvine, CA, USA). Concentrations were then determined using the Quant-iT™ High-Sensitivity DNA Assay Kit (Life Technologies). The sequencing reads and alpha diversity for negative controls were very low which were significantly different from the real samples, and therefore they are excluded from the analysis.

### Amplicon sequencing and data processing

Paired-end sequencing of the amplicon library was performed on the Illumina MiSeq System with MiSeq reagent kit v3 (2 × 300 bp, Illumina Inc., CA, USA), including 12.0% PhiX as an internal control. Demultiplexing in sample-specific raw fastq files were carried out directly on the MiSeq platform prior to downstream analysis. The identification of amplicon sequence variants (ASVs) was carried out on the QIIME 2 Core 2018.11 platform^75^ using DADA2^76^. After removing low-quality reads, a total of 1,292,378 high-quality sequences were obtained with a median read count per sample of 25,033 (range: 11,785–47,125). These high-quality reads were further identified into 9813 microbial ASVs based on single-base difference, which were affiliated to 32 phyla, 81 classes, 210 orders, 377 families and 788 genera, based on QIIME2 *classify-sklearn* function using SILVA 132 as a reference database^77^.

### Identification of the four strains’ specific ASVs in rhizo-microbiome

All the ASVs investigated in this study were aligned with the referred four species full-length 16S rRNA gene sequences that were uploaded in NCBI GenBank database in our previous study^78^ (accession numbers are Sr: JQ890538; Xr: JQ890537; Mo: JQ890539; Pa: JQ890540) by blastn^79^ to identify specific ASVs of each strain in the four-species consortium. It turned out that a unique ASV affiliated to that genus level of each strain was mapped successfully with 100% coverage and 100% identity against the referred full 16S rRNA gene sequences.

### Microscopy

To view adherent SPMX cells and multispecies biofilm formed on the root surface by confocal scanning laser microscopy (CLSM), the roots were stained at a dilution of 1:1000 with SYTO9 (Invitrogen) and 0.1% calcofluor white (CFW, Sigma-Aldrich). Roots with biofilm were captured by CLSM (LSM 800, Zeiss) with a Plan-Apochromat 63×/1.4 oil DIC M27. *Z*-stacks were recorded using Axiocam 503 mono to obtain three-dimension images. The maximum excitation and emission wavelengths for SYTO9 and CFW were 485 and 498 nm, 405 and 433 nm, respectively. Plants grown for 5 days in vitro on MS agar medium plates in the growth chamber were directly root-treated with 10 μl SPMX suspension (OD_600_ = 0.2 in TSB medium) by pipette, and co-cultivated in the growth chamber (150 μmol m^−2^ s^−1^ light intensity, 70% humidity, 12/12 h light/dark photoperiod with 22 °C/21 °C for daytime/night). Images were acquired 4 days of post co-cultivated with SPMX cells. A representative image for root colonization of SPMX was presented.

### Statistical analysis

Statistical analyses for plant physiological experiments were performed using SAS software. Data are presented as mean ± standard deviation (SD). Differences between two groups were analyzed by Student’s *t*-test. Multiple comparisons were analyzed by one-way analysis of variance (ANOVA) via Tukey’s studentized range (HSD) test (*P < 0.05, **P < 0.01, and ***P < 0.001).

The statistical analyses and data treatment for sequencing were carried out with the open-source statistical program “R”^80^, mainly in the R-package “phyloseq”^81^. Microbial alpha diversities between groups were compared using analysis of variance (R function “ano” in R package “stats”) on Shannon’s diversity index (H′). Microbial beta diversities between groups (over two groups) were compared by pairwise permutational multivariate analysis of variance (R function “pairwise.perm.manova” in R package “RVAideMemoire”^82^) with false discovery rate (FDR) correction on Bray–Curtis dissimilarity computations. Redundancy analysis by R function “rda” in the “vegan” package^83^ was used to test the effects of different environmental variables on microbial compositions. Hellinger transformation of the microbial relative abundances was used with RDA analysis. The explained variance R2 for each factor was adjusted by the R function “RsquareAdj” in “vegan” package and its statistical significance was tested by the Permutation test (R function “anova” in R package “vegan”). Random forest analysis was used to quantify the importance of the bacteria at various taxonomic levels in different groupings (R package “randomForest”^84^). Differentially abundant bacteria in two groups were detected using Wilcoxon rank-sum test and all obtained *P* values were corrected by FDR.

### Reporting summary

Further information on research design is available in the Nature Research Reporting Summary linked to this article.

## Supplementary information

Supplementary information

Reporting Summary

## Data Availability

Raw sequence data for all samples in this study have been deposited in the ENA-EBI with BioProject under accession number PRJEB40703. Other data that support the findings of this study within the article and Supplementary Information are available from the corresponding author upon reasonable request.

## Code availability

The key codes for the analyses by R in this study are available in Zenodo (https://zenodo.org/record/4312969#.YR5mBS8Rq3c).
