# Supplementary material for: Emergent bacterial community properties induce enhanced drought tolerance in Arabidopsis
Source: NPJ Biofilms Microbiomes. 2021 Nov 18;7:82. doi: 10.1038/s41522-021-00253-0 (PMC8602335; doi:10.1038/s41522-021-00253-0)
Supplement: Supplementary file 1 — Supplementary information [file 41522_2021_253_MOESM1_ESM.pdf]

Emergent bacterial community properties induce enhanced drought tolerance in *Arabidopsis*

Supplementary information

Supplementary Tables

Supplementary Table 1. List of primers used for quantitative RT-PCR.

Gene	Brief description	Primer	Sequence (5' - 3')	Reference
ACT2	Actin gene, inner expression control	For	AGTGTCTGGATCGGTGGTTC	(1)
		Rev	CCCCAGCTTTTTAAGCCTTT	
COR15A	Cold-regulated protein 15A, accumulating in response to ABA	For	CTCTGCCGCCTTGTTTGC	(2, 3)
		Rev	CTGAGAAAGCTGCGGCGTA	
NCED3	The enzyme NINE-CIS-EPOXYCAROTENOID DIOXYGENASE 3 (NCED3) essential for ABA biosynthesis during drought stress, and the *NCED3* gene is highly induced by drought stress.	For	GCTGCGGTTTCTGGGAGAT	(4, 5)
		Rev	GTCGGAGCTTTGAGAAGACGAT	
RAB18	Belonging to the dehydrin protein family, response to ABA, response to water deprivation	For	TCCAGCAGCAGTATGACGAGTA	(6)
		Rev	CCAGTTCCAAAGCCTTCAGTC	
TSPO	Outer membrane tryptophan-rich sensory protein, response to ABA, response to drought and salt stress	For	ACAAAGGAAAACGCGATCAAA	(7, 8)
		Rev	ACTTGAGACCACGTTTCGCC	

Supplementary Table 2. Description of Plug og såjord soil.

Description	Standard	Description	Standard
Degree of atomization:	Fine (NS 2890)	Degree of humification:	H2-H5 (von Post)
Peat dry matter:	approx. 110 kg / m^3, of which organic dry matter at least 95%	Reaction number:	pH 5.5-6.5 (LS 1965-5: 14-7)
Conductivity mS / cm:	1.5-2.5 (SLK M1-7 64 IV: 3)		
**Nutrient**	**Content (g / m$^{3)}$**	**Nutrient**	**Content (g / m$^{3)}$**
Nitrogen (N)	98	Phosphorus (P)	49
Potassium (K)	104	Magnesium (Mg)	247
Sulfur (S)	53	Calcium (Ca)	2186
Iron (Fe)	0.6	Manganese (Mn)	1.1
Copper (Cu)	0.8	Zinc (Zn)	0.3
Boron (B)	0.2	Molybdenum (Mo)	1.4

Supplementary Figures

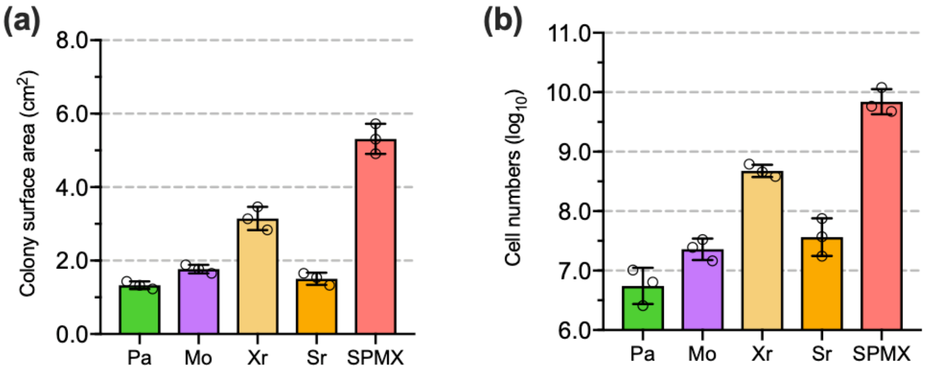

Supplementary Figure 1. Synergistic effects on biofilm among strain Sr, Pa, Mo and Xr in cocultures. Colony biofilm area (**a**) and total cell numbers of planktonic fraction (**b**) in four-species SPMX co-culture and its single-species culture Pa, Mo, Xr and Sr were estimated after 4-day and 48-hour incubation at 25 °C, respectively. Assays were performed three times. Error bars represent means ± s.d. for three replicates (n = 3).

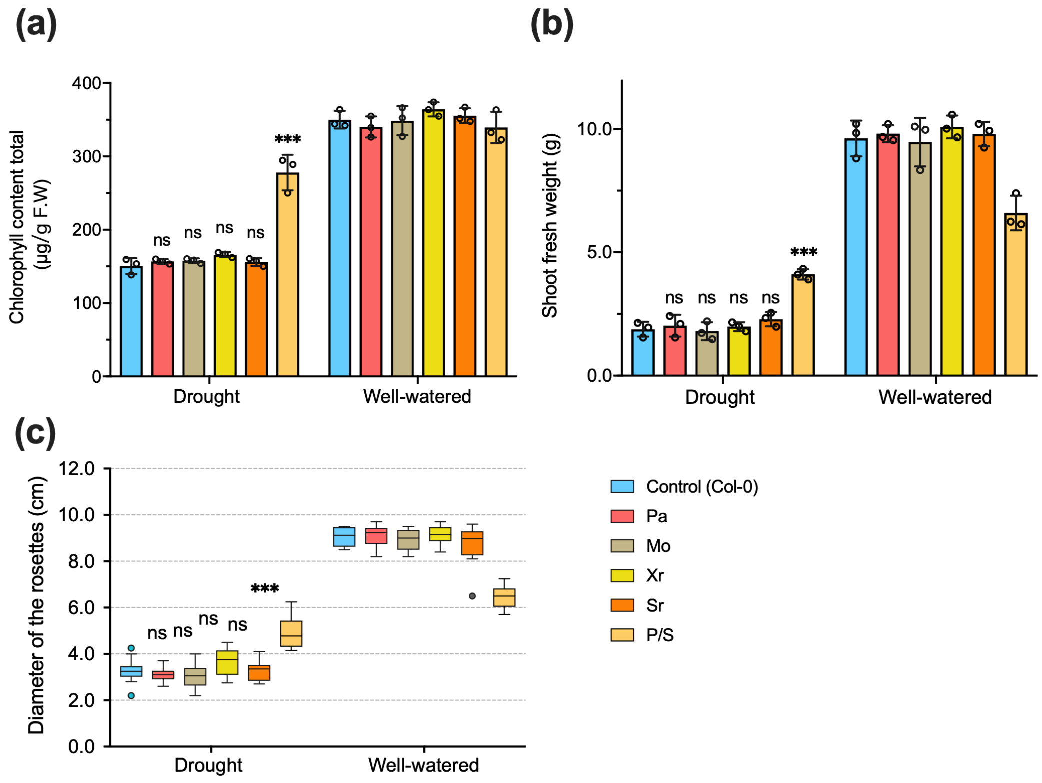

Supplementary Figure 2. The effect of inoculation with each individual strain of SPMX on plants growth. (**a**) total chlorophylls content (μg/g fresh weight) (n = 3 independent experiments); (**b**) shoot fresh weight (g) (n = 3 independent experiments) and (**c**) rosettes diameter (cm) under Fresh Weight (F.W) (n

= 10 replicates from three independent experiments) after 21 dpi either drought or watering treatment. Error bars represent mean ± standard deviation in (**a**) and (**b**). Asterisks above histograms are used to establish whether two group percentage values (different treatment VS control) under drought conditions are statistically significantly different as assessed by one-way ANOVA followed by a Tukey HSD test: *** $P < 0.001$. (ns= no statistical difference)

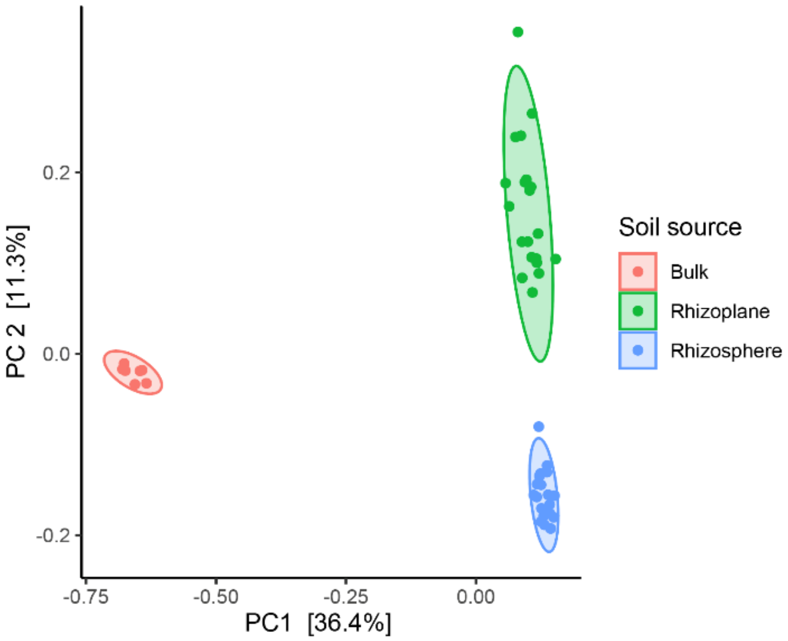

Supplementary Figure 3. Principal coordinates analysis (PCoA) based on Bray-Curtis dissimilarity matrix of the root microbiome composition. Samples are colored for soil source (bulk, rhizosphere and rhizoplane).

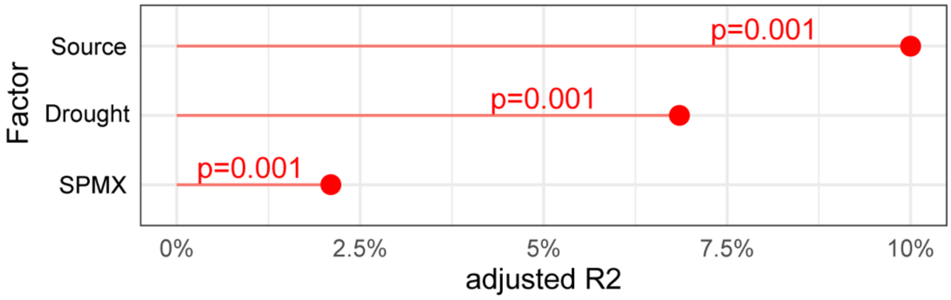

Supplementary Figure 4. Percent of variance explained by experimental factors (soil source, drought treatment and SPMX-inoculated treatment). The percent of these three factors are determined by distance based on redundancy analysis (db-RDA) using the Bray–Curtis dissimilarity matrix of the root

microbiome composition. The x-axis indicates the fraction of variance explained, and the *P*-value indicates the significance (Pairwise PERMANOVA).

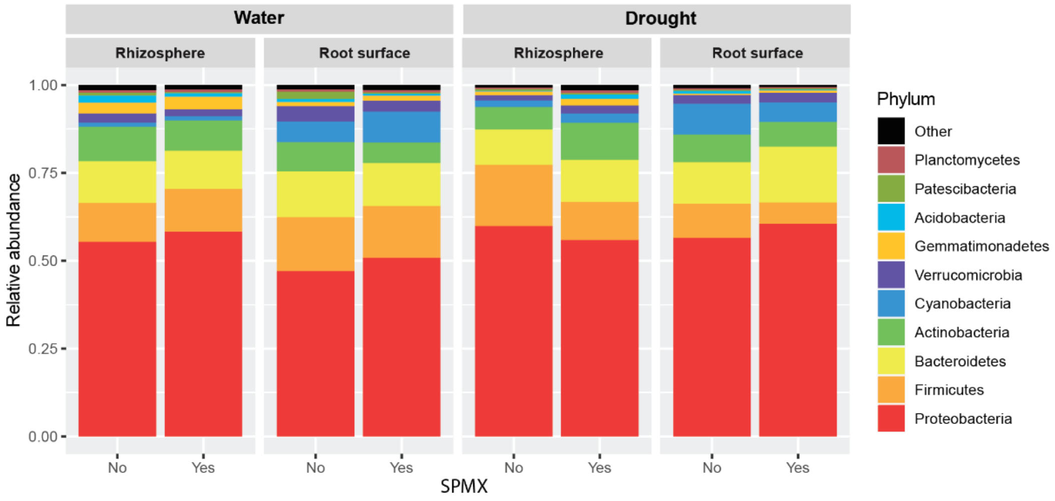

Supplementary Figure 5. The relative abundance of the top ten most abundant bacterial phyla in different combinations between soil compartment (rhizosphere/rhizoplane) and treatments (water/drought; SPMX/non-SPMX). Data based on five samples (n = 5) collected in each treated group

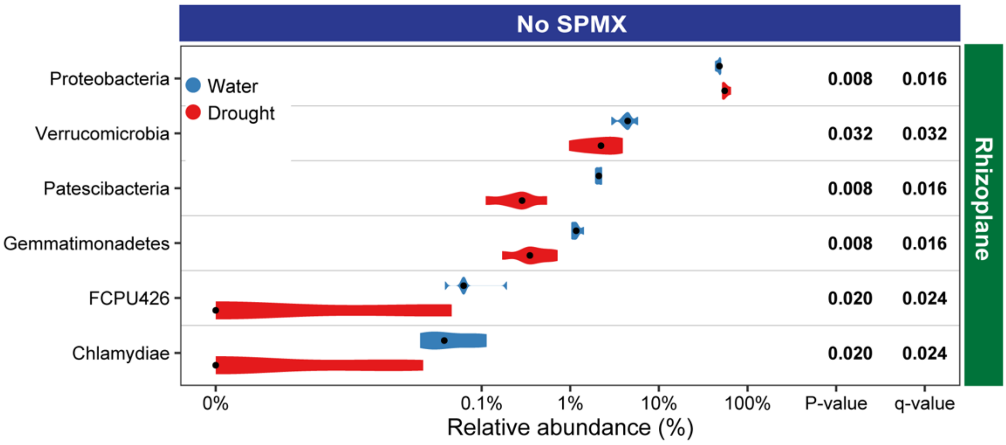

Supplementary Figure 6. Violin plots showing the significantly differentially abundant bacterial phyla between drought (red) and watered (blue) rhizoplane samples in absence of SPMX. All of statistically significant values ($P < 0.05$ after FDR adjustment, Wilcoxon rank-sum test) were shown. Data based on five samples (n = 5) collected in each treated group

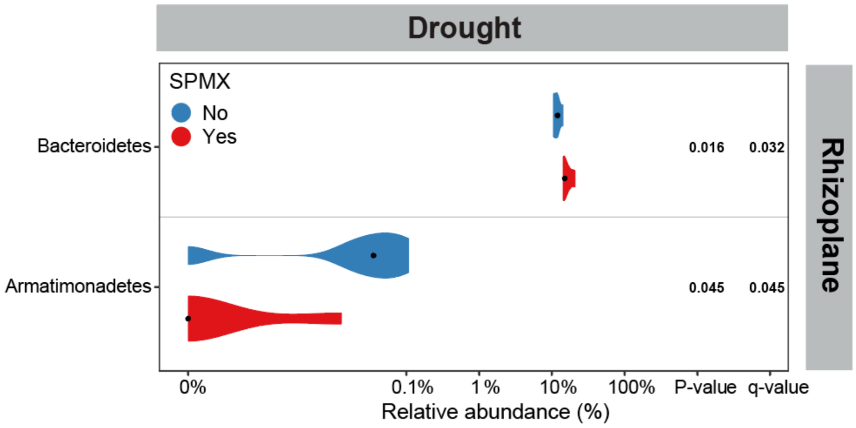

Supplementary Figure 7. Violin plots showing the significantly differentially abundant bacterial phyla between SPMX inoculated (red) and non-SPMX inoculated (blue) soils in the rhizoplane under drought conditions. All of statistically significant values (*P* < 0.05 after FDR adjustment, Wilcoxon rank-sum test) were shown. Data based on five samples (n = 5) collected in each treated group

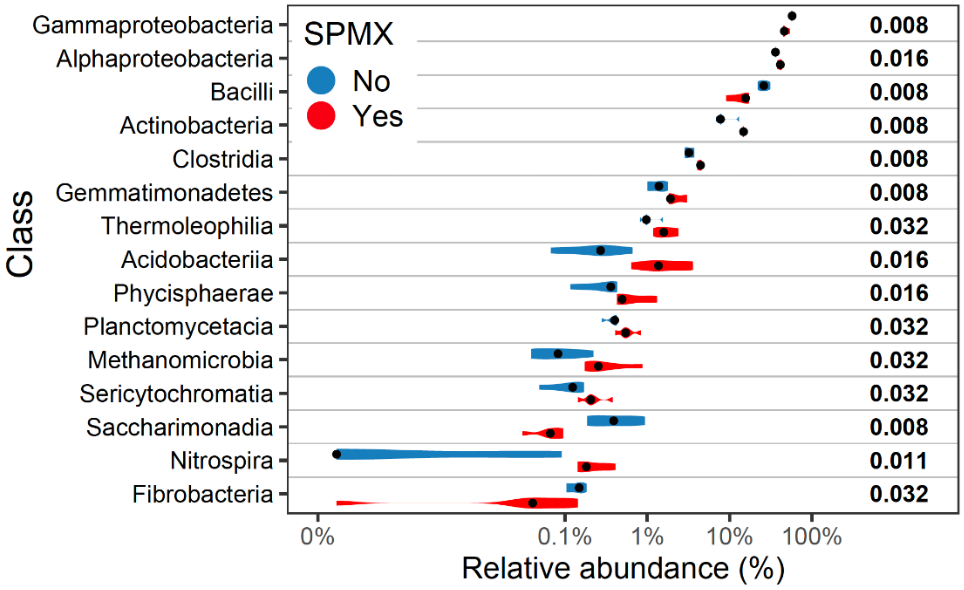

Supplementary Figure 8. Significantly differential bacterial classes in the relative abundance between SPMX inoculated (red) and non-SPMX inoculated (blue) samples in the rhizosphere under drought. All of the statistically significant values (FDR adjusted P < 0.05, Wilcoxon rank-sum test) were shown. Data based on five samples (n = 5) collected in each treated group

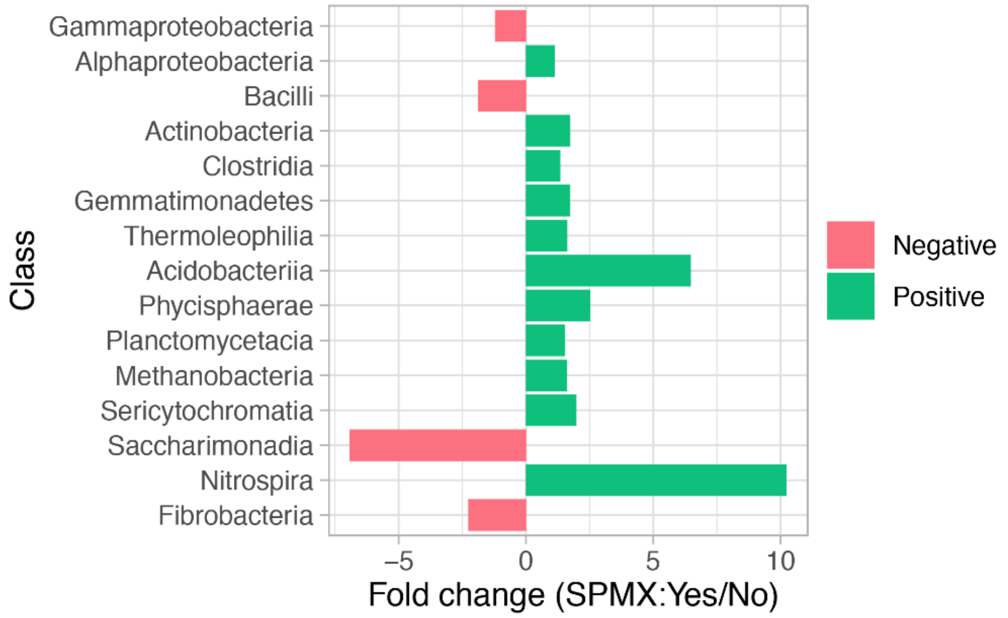

Supplementary Figure 9. The fold changes of significantly changed bacterial classes in the relative abundance between SPMX-inoculated and non-SPMX inoculated samples (SPMX: Yes/No) in the rhizosphere under drought conditions. Red color represents negative change and green color represents positive change.

References

1. Y. Q. An *et al.*, Strong, constitutive expression of the Arabidopsis ACT2/ACT8 actin subclass in vegetative tissues. *Plant J* **10**, 107-121 (1996).
2. Y. Zhu *et al.*, CDK8 is associated with RAP2. 6 and SnRK2. 6 and positively modulates abscisic acid signaling and drought response in Arabidopsis. *The New Phytologist* (2020).
3. N. N. Artus *et al.*, Constitutive expression of the cold-regulated Arabidopsis thaliana COR15a gene affects both chloroplast and protoplast freezing tolerance. *Proc Natl Acad Sci U S A* **93**, 13404-13409 (1996).
4. S. Iuchi *et al.*, Regulation of drought tolerance by gene manipulation of 9-cis-epoxycarotenoid dioxygenase, a key enzyme in abscisic acid biosynthesis in Arabidopsis. *Plant J* **27**, 325-333 (2001).
5. H. Sato *et al.*, Arabidopsis thaliana NGATHA1 transcription factor induces ABA biosynthesis by activating NCED3 gene during dehydration stress. *P Natl Acad Sci USA* **115**, E11178-E11187 (2018).
6. V. Lang, E. T. Palva, The Expression of a Rab-Related Gene, Rab18, Is Induced by Abscisic-Acid during the Cold-Acclimation Process of Arabidopsis-Thaliana (L) Heynh. *Plant Mol Biol* **20**, 951-962 (1992).
7. C. Hachez *et al.*, The Arabidopsis Abiotic Stress-Induced TSPO-Related Protein Reduces Cell-Surface Expression of the Aquaporin PIP2;7 through Protein-Protein

Interactions and Autophagic Degradation. *Plant Cell* **26**, 4974-4990 (2014).

8. D. Guillaumot *et al.*, The Arabidopsis TSPO-related protein is a stress and abscisic acid-regulated, endoplasmic reticulum-Golgi-localized membrane protein. *Plant J* **60**, 242-256 (2009).
