# Supplementary material for: Emergent bacterial community properties induce enhanced drought tolerance in Arabidopsis
Source: NPJ Biofilms Microbiomes. 2021 Nov 18;7:82. doi: 10.1038/s41522-021-00253-0 (PMC8602335; doi:10.1038/s41522-021-00253-0)
Supplement: Supplementary file 2 — Reporting Summary [file 41522_2021_253_MOESM2_ESM.pdf]

nature portfolio

Corresponding author(s): Søren J. Sørensen
Mette Burmølle

Last updated by author(s): Oct 5, 2021

Reporting Summary

Nature Portfolio wishes to improve the reproducibility of the work that we publish. This form provides structure for consistency and transparency in reporting. For further information on Nature Portfolio policies, see our Editorial Policies and the Editorial Policy Checklist.

Statistics

For all statistical analyses, confirm that the following items are present in the figure legend, table legend, main text, or Methods section.

n/a	Confirmed	
☐	☒	The exact sample size (*n*) for each experimental group/condition, given as a discrete number and unit of measurement
☐	☒	A statement on whether measurements were taken from distinct samples or whether the same sample was measured repeatedly
☐	☒	The statistical test(s) used AND whether they are one- or two-sided<br>*Only common tests should be described solely by name; describe more complex techniques in the Methods section.*
☒	☐	A description of all covariates tested
☒	☐	A description of any assumptions or corrections, such as tests of normality and adjustment for multiple comparisons
☐	☒	A full description of the statistical parameters including central tendency (e.g. means) or other basic estimates (e.g. regression coefficient) AND variation (e.g. standard deviation) or associated estimates of uncertainty (e.g. confidence intervals)
☐	☒	For null hypothesis testing, the test statistic (e.g. *F*, *t*, *r*) with confidence intervals, effect sizes, degrees of freedom and *P* value noted<br>*Give P values as exact values whenever suitable.*
☒	☐	For Bayesian analysis, information on the choice of priors and Markov chain Monte Carlo settings
☒	☐	For hierarchical and complex designs, identification of the appropriate level for tests and full reporting of outcomes
☒	☐	Estimates of effect sizes (e.g. Cohen's *d*, Pearson's *r*), indicating how they were calculated

Our web collection on statistics for biologists contains articles on many of the points above.

Software and code

Policy information about availability of computer code

Data collection	R version 4.0.2
Data analysis	Analysis code is available at: https://doi.org/10.5281/zenodo.4312969.<br><br>The statistical analyses and data treatment for sequencing were carried out with the open-source statistical program “R”, mainly in the R-package “phyloseq”. Microbial alpha diversities between groups were compared using analysis of variance (R function “ano” in R package “stats”) on Shannon’s diversity index (H’). Microbial beta diversities between groups (over 2 groups) were compared by pairwise permutational multivariate analysis of variance (R function “pairwise.perm.manova” in R package “RVAideMemoire”) with false discovery rate (FDR) correction on Bray Curtis dissimilarity computations. Redundancy analysis by R function “rda” in the “vegan” package was used to test the effects of different environmental variables on microbial compositions. Hellinger transformation of the microbial relative abundances was used with RDA analysis. The explained variance R2 for each factor was adjusted by the R function “RsquareAdj” in “vegan” package and its statistical significance was tested by the Permutation test (R function “anova” in R package “vegan”). Random forest analysis was used to quantify the importance of the bacteria at various taxonomic levels in different groupings (R package “randomForest”). Differentially abundant bacteria in two groups were detected using Wilcoxon rank-sum test and all obtained P values were corrected by FDR.

For manuscripts utilizing custom algorithms or software that are central to the research but not yet described in published literature, software must be made available to editors and reviewers. We strongly encourage code deposition in a community repository (e.g. GitHub). See the Nature Portfolio guidelines for submitting code & software for further information.

Data

Policy information about availability of data

All manuscripts must include a data availability statement. This statement should provide the following information, where applicable:

- Accession codes, unique identifiers, or web links for publicly available datasets
- A description of any restrictions on data availability
- For clinical datasets or third party data, please ensure that the statement adheres to our policy

All sequence data acquired in this study are available at ENA-SRA database under BioProject accession number PRJEB40703

Field-specific reporting

Please select the one below that is the best fit for your research. If you are not sure, read the appropriate sections before making your selection.

☒ Life sciences ☐ Behavioural & social sciences ☐ Ecological, evolutionary & environmental sciences

For a reference copy of the document with all sections, see nature.com/documents/nr-reporting-summary-flat.pdf

Life sciences study design

All studies must disclose on these points even when the disclosure is negative.

Sample size	No sample size estimation was conducted. We used five independent experiments, each including all conditions, to test drought survival. Each sample groups had a minimum of 20 individual plants in minimum 5 pots and up to 32 individual plants in 8 pots (depending on available germinated seeds at the beginning of the experiment)
Data exclusions	No data was excluded.
Replication	We used five independent experiments to assess the reproducibility of the observed drought resistance. Amplicon sequencing was performed on five individual plants per treatment randomly chosen from each group.
Randomization	All pots were organized in a completely random design in the climate chamber.
Blinding	Blinding was not relevant for this study: all output was measured at end point and treatments (watering/bacterial inoculation) allocated from the start.

Reporting for specific materials, systems and methods

We require information from authors about some types of materials, experimental systems and methods used in many studies. Here, indicate whether each material, system or method listed is relevant to your study. If you are not sure if a list item applies to your research, read the appropriate section before selecting a response.

Materials & experimental systems

n/a	Involved in the study	
☒	☐	Antibodies
☒	☐	Eukaryotic cell lines
☒	☐	Palaeontology and archaeology
☐	☒	Animals and other organisms
☒	☐	Human research participants
☒	☐	Clinical data
☒	☐	Dual use research of concern

Methods

n/a	Involved in the study	
☒	☐	ChIP-seq
☒	☐	Flow cytometry
☒	☐	MRI-based neuroimaging

Animals and other organisms

Policy information about studies involving animals; ARRIVE guidelines recommended for reporting animal research

Laboratory animals	*For laboratory animals, report species, strain, sex and age OR state that the study did not involve laboratory animals.*
Wild animals	*Provide details on animals observed in or captured in the field; report species, sex and age where possible. Describe how animals were caught and transported and what happened to captive animals after the study (if killed, explain why and describe method; if released, say where and when) OR state that the study did not involve wild animals.*

Field-collected samples	That study did not involve any field samples. We used archetypal Columbia (Col-0) strain of Arabidopsis thaliana and a double knock-out mutant obtained from co-author Morten Petersen.
Ethics oversight	*Identify the organization(s) that approved or provided guidance on the study protocol, OR state that no ethical approval or guidance was required and explain why not.*

Note that full information on the approval of the study protocol must also be provided in the manuscript.
